# Animated Videos Based on Food Processing for Guidance of Brazilian Adults: Validation Study

**DOI:** 10.2196/49092

**Published:** 2023-09-11

**Authors:** Maria Fernanda Gomes da Silva, Luciana Neri Nobre, Edson da Silva

**Affiliations:** 1 Graduate Program in Nutrition Sciences Federal University of Jequitinhonha and Mucuri Valleys Diamantina Brazil; 2 Department of Nutrition Federal University of Jequitinhonha and Mucuri Valleys Diamantina Brazil; 3 Department of Basic Sciences Federal University of Jequitinhonha and Mucuri Valleys Diamantina Brazil

**Keywords:** food, processed, dietary guidelines, nutrition policy, instructional films and videos, validation study, food classification, validation, educational videos, nutrition, Brazil

## Abstract

**Background:**

Ultraprocessed foods (UPFs) contribute almost one-fifth of the calories consumed by the Brazilian population. This consumption has been favored by aspects such as the ease of acquisition and low cost of this food group. Initiatives focused on supporting and promoting healthy eating practices have been implemented. Among them, the availability of educational resources is an important strategy to maximize the effectiveness of these actions in the field of food and nutrition education (FNE).

**Objective:**

This study aims to describe the development and validation process of animated videos based on the NOVA food classification for FNE actions aimed at Brazilian adults.

**Methods:**

This methodological study was developed in the following 4 phases: planning, preproduction, production, and postproduction. In the planning phase, a literature review was con-ducted on the topic and to define the content to be covered. The design of the material was based on the cognitive theory of multimedia learning. In the preproduction phase, video scripts were developed and evaluated by 7 content specialists. In the production phase, videos were developed based on the assessed scripts and then assessed by 3 multimedia production specialists. In the postproduction phase, the videos were evaluated by 15 representatives of the target audience. All results obtained in the evaluation phases were analyzed using the content validity index (CVI).

**Results:**

We developed 3 animated videos covering the following themes: food processing levels, food categories according to processing levels, and UPFs and their impact on health. In the evaluation by the content specialists, the scripts of videos 1, 2, and 3 obtained CVIs at the scale level and average method equal to 0.96, 0.98, and 0.98, respectively. When the animated videos were evaluated by multimedia production specialists and representatives of the target audience, these indexes were equal to 1.0. These results attest to the videos’ adequacy and quality in communicating the addressed content.

**Conclusions:**

The animated videos developed and validated in this study proved to be adequate for their purpose. Thus, it is expected that they will be an important instrument for FNE actions aimed at an adult audience and for disseminating the Dietary Guidelines for the Brazilian Population.

## Introduction

Eating patterns are changing rapidly in the vast majority of countries, particularly in middle-income countries. The main changes in this nutritional transition involve the replacement of culinary preparations based on in natura or minimally processed foods with ultraprocessed foods (UPFs) [1].

In Brazil, according to data from the Surveillance of Risk and Protective Factors for Chronic Diseases by Telephone Inquiry in 2019 [2], UPF already contributed almost one-fifth of the calories consumed, especially from salty cookies and packaged snacks, industrialized bread, sweet cookies, and cold cuts and sausages. A survey carried out by Monteiro and collaborators [3] pointed out that, in middle-income countries, the average growth in sales of these products reached approximately 10%.

This pattern of consumption has led to an imbalance in the supply of nutrients and excessive intake of calories [1]. A recent study showed that, in 2019, UPF consumption was responsible for approximately 57,000 deaths in Brazil, corresponding to 10.5% of all premature deaths and 21.8% of all preventable deaths from chronic noncommunicable diseases (NCDs) in adults aged 30 years to 69 years [4].

Aspects such as the greater ease of purchasing UPFs and their low cost and high energy density have contributed to the increase in consumption of these foods, as well as to the current scenario of NCDs like obesity, diabetes, and hypertension, which in turn are associated with a large economic burden for the Unified Health System in Brazil [3-6]. Another concern is associated with equity, given that UPF consumption has increased more rapidly among low-income households [4].

However, despite the understanding that a balanced diet is an important factor for maintaining health, healthy eating practices encompass not only the foods that are commonly consumed but also cultural factors, ways of life, food availability, income, health literacy, marketing, and media, among other aspects [7]. Thus, food and nutrition education (FNE) has been recognized as a strategy for the prevention and control of food and nutrition problems that incorporates sociocultural, biological, and environmental dimensions in a transdisciplinary, intersectoral, and multiprofessional field [7].

In this practice, the Dietary Guidelines for the Brazilian Population (DGBP) [1] is the official Brazilian document that brings together recommendations for healthy eating practices and is configured as an instrument to support FNE actions. The golden rule, “always prefer *in natura* or minimally processed foods and culinary preparations to ultraprocessed foods,” is the main recommendation of the DGBP and is based on the NOVA classification system, which groups foods according to the extent and purpose of the processing they have undergone into the following 4 groups: (1) unprocessed and minimally processed foods, (2) processed culinary ingredients, (3) processed foods, and (4) UPFs [1,3].

NOVA not only influenced the foundation of the DGBP but also guided its implementation in public nutrition and health policies in Brazil and has been increasingly favorable for the epidemiological and food culture fields [8]. Strategies including development of protocols and instructions have played an important role in qualifying health service professionals for this purpose [6,8]. However, educational actions demand educational resources that can promote equitable access and engagement that meet the different needs and levels of health literacy of individuals, families, and communities [9].

The rise of online communication has expanded possibilities to make health communication more attractive [10]. In addition, educational animations, when properly produced, have been seen as a positive strategy to communicate complex health messages [11], and the adoption of digital resources has been associated with the strengthening of the interface between communication, science, and society as a consequence of aspects related to motivation, dynamics, transversality, and the absence of geographic barriers [12,13].

The elaboration of educational materials is part of a planned strategy for the implementation of the DGBP. Developing and validating educational videos for application in FNE actions can contribute to communication effectiveness and dissemination of knowledge among professionals and society. However, until now, the creation of videos has not been described in published, structured studies. Therefore, the purpose of this study was to develop and validate a series of 3 animated videos to support educational actions aimed at Brazilian adults.

## Methods

### Study Types

This is a descriptive study of the development and validation of 3 animated videos based on the DGBP aimed at Brazilian adults.

**Figure 1:**
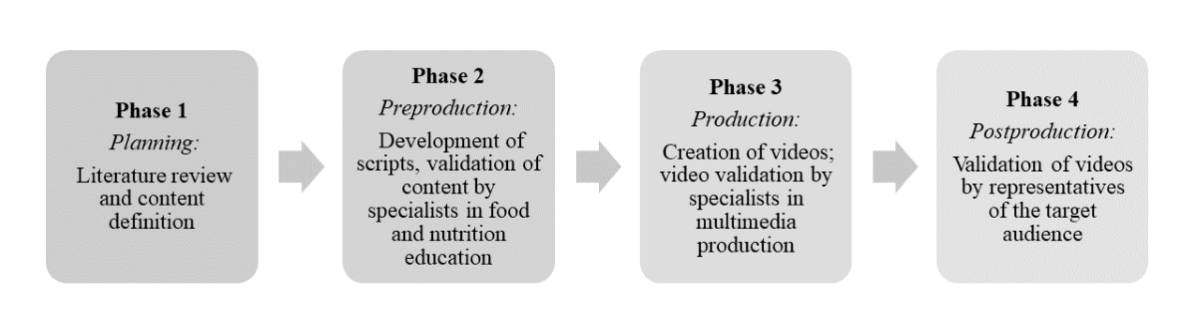
Animated video development and validation process.

The development of the videos was based on the stages of animation production proposed by Winder and Dowlatabadi [14] and Wright [15] and occurred in the following 4 phases: planning, preproduction, production, and postproduction (Figure 1).

### Video Development and Validation Process

#### Phase 1: Planning

A search was conducted in the scientific databases PubMed, Literatura Latino-Americana e do Caribe em Ciências da Saúde (LILACS), and Scientific Electronic Library Online (Scielo) with the objective of identifying studies and audiovisual educational materials aimed at the Brazilian adult population (18-59 years), based on food processing and DGBP, analyzing methodologies, limitations and possible advances for this proposal, and YouTube videos provided online. The searches in the databases were performed for articles published from 2014 to 2021, by combining the following descriptors (and their corresponding terms in Portuguese): “Food Guides” AND “Educational Film and Video” OR “Educational Technology.” On YouTube, this search was made using the terms “Food Guide” and “NOVA Classification.”

Next, the team that conducted the study applied the technique of brainstorming to collect and select ideas related to the topic and suitable for the target audience that the material would address. On this basis, we decided to draw on the content of Chapter 2 of the DGBP, “The choice of food,” and the works published by Monteiro and collaborators [3,16], publications that are more recent than the NOVA classification.

This material was divided into 3 animated videos and planned to be used in FNE actions in a segmented way. The design was based on the principles and guidelines of the Framework of Reference for Food and Nutrition Education [7] and the applications of the cognitive theory of multimedia learning (CTML) [17].

The decision to develop and validate videos was based on an understanding of the 3 assumptions of CTML: (1) 2 channels (visual and auditory) for receiving and processing information, (2) limited capacity of information that can be processed in each channel, and (3) learning as an active process that involves organizing and integrating previous information into the most recent [17].

According to Mayer [17], the design of multimedia environments must be compatible with the way people learn. Therefore, the 12 didactic principles were considered in the design of the developed material: Coherence Principle (exclusion of unnecessary words, symbols, images, and sounds), Signaling Principle (highlighting key elements of the content covered), Redundancy Principle (using animation and narration instead of animation, narration, and text on the screen), Spatial Contiguity Principle (using pictograms and texts in spatial proximity), Temporal Contiguity Principle (simultaneously presenting pictograms and narration), Segmentation Principle (presenting the content in segments), Pre-training Principle (general presentation of the content before detailing it), Modality Principle (use of pictograms and narration instead of pictograms and text), Personalization Principle (use of conversational style words instead of formal style), Voice Principle (use of the narration in human voice), and Image Principle (the image of the narrator is dispensable) [17].

#### Phase 2: Preproduction

The central ideas for each video were planned to be approached in a sequential order seeking to deepen the theme of the previous media. Thus, a narrative text was developed for each material, taking this proposal into account. One of the premises for the material development was the method of approaching the content. To this end, the language of the material was planned and revised with the aim of minimizing the cognitive load of learning and allowing the target audience to better understand the topic. Then, each narrative text was divided into scenes, which were then described and outlined.

In the subsequent step, *storyboards* were developed, then a script for each video was structured in the following 3 parts: narration, scene description, and storyboard.

After the scripts were completed, the content was validated by specialists. At this stage, nutrition professionals were invited and selected for convenience. For the selection of these judges, knowledge and level of expertise were considered [18], adopting criteria from an existing model commonly used in validation studies in Brazil [19] that assigns a score for the judge's experience in the topic of interest. In this study, experience in FNE and DGBP was the considered criterion, as evidenced by participation in research, analysis of publications on the subject, and teaching in disciplines in the area. Having a master's degree was a minimum criterion. The identification of these specialists occurred through publications and searches in research programs of Brazilian universities. Professionals who obtained a minimum score of 5 points through curriculum analysis were considered able to evaluate the scripts [19].

After the selection of potential judges, the invitation to participate in the study was sent by email, which included a link to access a form developed in Google Forms with the informed consent form, a questionnaire to characterize the specialists’ profiles, and the Educational Content Validation Instrument in Health (ECVIH; Multimedia Appendix 1) [20], as well as a blank space for suggestions to improve the material. The recruitment of judges was completed when the evaluation of 7 specialists was complete, with the panel defined by the recommendation that evaluation by at least 5 judges can provide a sufficient level of control for casual agreement [21]. This evaluation process took place from April 2022 to May 2022.

#### Phase 3: Production of Animated Vídeos

After the script was evaluated by the content specialists, the requested suggestions and adjustments were analyzed, including adding or replacing examples and rewording excerpts from the narratives to make them more understandable.

Once the adjustments were completed, the narratives for each video were recorded. Based on the storyboards, the scenes were developed on the Canva platform. For this purpose, a mood board was first created with videos, photos, and pictograms. Based on this, each scene was created following the flipbook concept of the platform resources, gradually changing the position of the photos and pictograms if necessary. The entire sequence of images created was transferred to Movavi Video Editor and sequenced to create the impression of motion.

With the first version of each video, an evaluation of the appropriateness of the material was carried out by multimedia production specialists. The identification of these experts occurred via LinkedIn, and the selection took place by analyzing the curriculum. To be considered able to evaluate the videos, we selected those who obtained a minimum score of 5 points in aspects that encompassed knowledge and experience in the production of animations [19].

These specialists were contacted, and the proposal was presented. For those who agreed to participate in the study, an email was sent with the same structure as in the previous step, but we used the Suitability Assessment of Materials (SAM) [22] tool, translated and adapted for use in Brazil [23] (Multimedia Appendix 2). The objective of the SAM is to evaluate the suitability of educational material. The recruitment was completed when the assessment of 3 professionals was complete, with the panel defined by the recommended minimum number [21]. The evaluation process took place between August 2022 and September 2022.

#### Phase 4: Postproduction

After the evaluation of the videos was completed, the dissonances between the sound and text were corrected, and the pictograms were changed according to the suggestions from the experts. There was also a proposal to unify the visual language of the video; however, this proposal could not be fully implemented because there are few pictograms with the same language in the graphic elements database of the Canva platform. Alternative platforms with public domain images were researched, but no substitute was found that fulfilled the same communication purpose.

With the adjustments made, the animations were evaluated by representatives of the target audience. The sample size was calculated using the formula for a finite population: n=Za2.P(1-P)/e2, where *Za* corresponds to the confidence level, *P* is the expected proportion of judges who agree with the item, and *e* is the expected difference. The following values were assumed for this purpose: Za=95%, *P*=90%, and e=15%. This resulted in 15 participants [24].

Through snowball sampling [25], the research team selected and invited participants through their social networks. Each guest could indicate 1 to 2 people from their social network, and the latter could indicate at least one other participant. The inclusion criteria were adults of both sexes aged between 18 years and 59 years who did not present with impairments in cognitive status, vision, and hearing. Recruitment was complete when 15 representatives of the target group were identified.

Each participant had access to an informed consent form provided on a form created with Google Forms and, after confirming his or her consent, was directed to a questionnaire collecting sociodemographic information (age, sex, race/color, education) and a self-report of diseases related to cognitive status, vision, and hearing adopted from population-based surveys in Brazil [2,26]. The assessment tool used in this phase was the SAM [23] (Multimedia Appendix 2). The evaluation process took place from September 2022 to October 2022.

### Data Analysis

The ratings by content specialists, multimedia production professionals, and representatives of the target audience were analyzed using the content validity index (CVI), which has the objective of measuring the percentage of evaluators who agree on the aspects analyzed in the instrument [27].

This index was calculated in the following 2 ways: (1) using the item-level content validity index (I-CVI), obtained by the sum of the judges' agreement on each item of the ECVIH and SAM instruments divided by the total number of responses, and (2) using the scale-level content validity index, average calculation method (S-CVI/Ave) to assess the average agreement of the judges, obtained by the sum of the I-IVC of each item divided by the total number of items of each instrument [27].

For general content validation, an index of 0.90 or higher was considered desirable for the S-CVI/Ave [28]. For the I-CVI, values no lower than 0.78 were considered [21]. Indexes that had lower values than those described were revised according to the experts' suggestions. All analyses were performed using Excel.

### Ethics Approval

The study was conducted in accordance with Resolution 466/2012 [29] of the Brazilian National Health Council. The study was approved by the Research Ethics Committee of the Federal University of Vales do Jequitinhonha e Mucuri (CAAE: 47624721.9.0000.5108).

## Results

In this study, 3 educational videos were developed based on the NOVA classification of foods [1,3,16]: food processing levels, food categories by processing levels, and UPFs and their effects on health.

**Figure 2:**
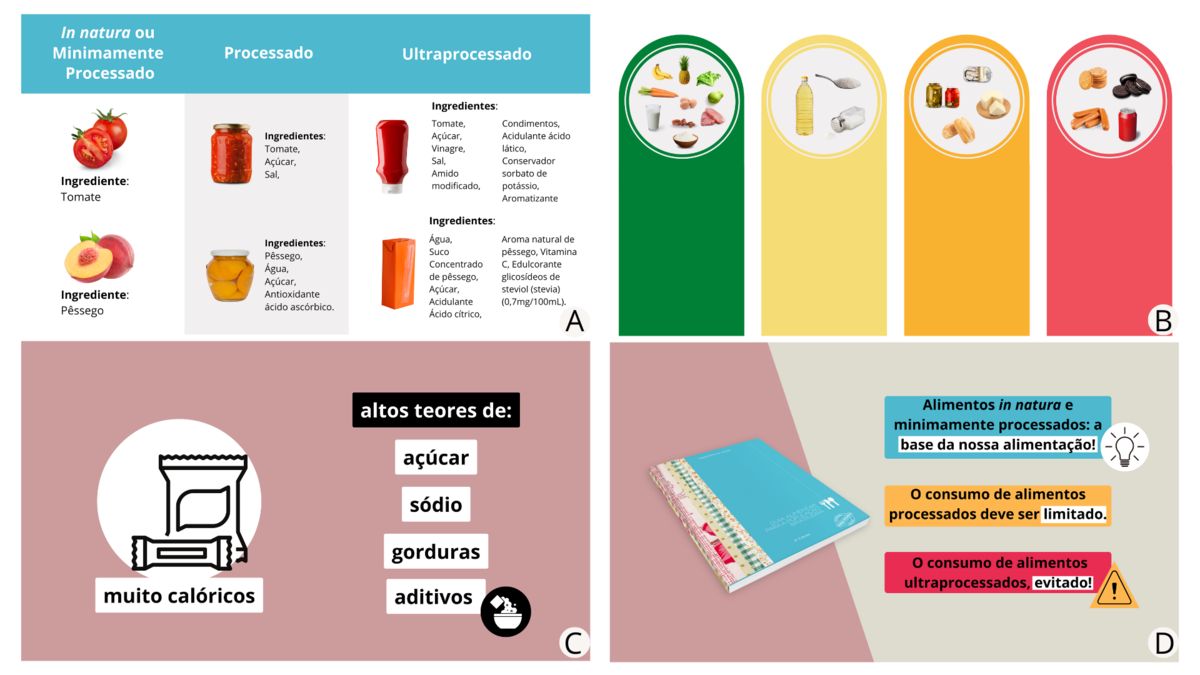
Scenes from (A) animated video 1, exemplifying the levels of processing; (B) animated video 2, showing food groups from the NOVA classification; (C) animated video 3, which cites some of the characteristics of ultra-processed foods ("high in calories, sugar, sodium, fat, and additives"); as well as (D) recommendations from the Food Guide for the Brazilian Population.

Animated video 1 is 3 minutes and 45 seconds long (Multimedia Appendix 3) and focuses on addressing the extent and purpose of food processing, introducing concepts, and situating the public on the subject addressed. Animated video 2 is 3 minutes and 25 seconds long (Multimedia Appendix 4) and provides deeper content on the main differences between the food groups in the NOVA classification system. Animated video 3 is 4 minutes and 12 seconds long (Multimedia Appendix 5) and highlights the group of UPFs only, describes the characteristics of the foods in this group, and reaffirms the importance of avoiding daily consumption of this group. Figure 2 shows scenes from the 3 videos. To arrive at the final result, each animated video was evaluated for its content and suitability as an educational resource for the target audience; the following sections describe how this entire process unfolded.

The stage involving validation of the script content involved 7 experts in FNE, of whom 57% (n=4) were doctors and 43% (n=3) had a master's degree. They were residents of 4 federative units and 3 different regions of the country. In terms of professional experience, 71% (5/7) had teaching experience in FNE, and 57% (4/7) reported having at least 10 years of practical experience in the area. All participants had research or work experience related to the NOVA and DGBP classification systems. Regarding scientific production, 86% (6/7) had published a scientific article in an indexed journal in the area of FNE.

The ECVIH [20], which was used to evaluate the scripts, has 18 items. From the analyzed results, the evaluation of the script referring to animated video 1 (S1) showed a mean overall agreement (S-CVI/Ave) of 0.96. For the script for animated video 2 (S2), this agreement was equal to 0.98, and for the script for animated video 3 (S3), it was 0.98 (Table 1). According to the criteria by Lynn [21], the values obtained for S-CVI/Ave indicate excellent content validity.

**Table 1:** FNE experts' agreement regarding the content of the scripts (n=7).

Item		I-CVI^a^		
		S1^b^	S2^c^	S3^d^
**Objectives:** **purposes, goals, or targets**				
	Contemplates the proposed theme	1	0.86	1
	Suits the teaching-learning process	1	1	1
	Clarifies doubts on the addressed theme	1	1	1
	Provides reflection on the theme	0.86	1	0.86
	Encourages behavior change	0.71	0.86	0.86
**Structure and presentation: organization, structure, strategy, consistency, and sufficiency**				
	Language appropriate to the target audience	1	1	1
	Language appropriate to the educational material	1	1	1
	Interactive language, enabling active involvement in the educational process	0.86	1	1
	Correct information	1	1	1
	Objective information	1	0.86	0.86
	Enlightening information	1	1	1
	Necessary informations	1	1	1
	Logical sequence of ideas	1	1	1
	Current theme	1	1	1
	Appropriate text size	0.86	1	1
**Relevance: significance, impact, motivation, and interest**				
	Encourages learning	1	1	1
	Contributes to knowledge in the area	1	1	1
	Arouses interest in the theme	1	1	1
S-CVI/Ave^e^		0.96	0.98	0.98

^a^I-CVI: item-level content validity index.

^b^S1: script 1.

^c^S2: script 2.

^d^S3: script 3.

^e^S-CVI/Ave: scale-level content validity index, averaging method.

When analyzing the I-CVI results obtained for each script (Table 1), for S1, 14 items presented an I-CVI of 1.00, and 3 had an index equal to 0.86, results that prove the good quality of the produced content. Relevant items for the development of the material, especially regarding the language and content, obtained an excellent evaluation. The item “Encourages behavior change” referring to the “Objectives” domain, however, obtained a lower score (I-CVI=0.71), being judged as inadequate by 2 of the 7 judges. Following the observation made by Lynn [21], when 6 or more experts carry out an evaluation, if one or more experts disagree with the others, what must be evaluated is content validity.

A suggestion for this script was the inclusion of activities for reflection, a phase already foreseen in the research but not described in this work. In addition, adjustments were made to the beginning and end of the script to generate greater interest and engagement among the target audience as an incentive for change.

In S2 and S3, 15 items had an I-CVI of 1.00, and 3 had an index of 0.86. For S2, one juror suggested that the title of the video be adjusted, justifying that interest in the topic might decrease if terms included in the name of the video were not familiar. To this end, the title “Food categories in the NOVA classification” was changed to “Food classification by processing levels.“ Regarding S3, suggestions for adjustments in certain parts of the text were adopted to avoid confusion of terms. Further comments have been inserted in Table 2.

**Table 2:** Experts' main comments and suggestions regarding general aspects of the scripts.

Script number	Comments and suggestions
Script 1	When the script addresses ultraprocessing: ”I suggest valuing that processing is not possible at home, that it alters the fresh food so much that it is not possible to recognize it.“ (Judge 3) ”I suggest including some activity proposals to stimulate reflection, for example: ‘Try to classify the food you have at home/How do you evaluate your diet after this check?’“ (Judge 6) ”I found that some texts were a little long, but I don't see any major problems since it will be narrated.“ (Judge 7)
Script 2	”The recommendations of the guide were cited in all groups but the ultraprocessed ones: 'Avoid ultraprocessed foods.’ Include to standardize and emphasize.” (Similar comments between Judges 2 and 5) “I suggest inserting a scene explaining the label and including some questions that lead the audience to look for a label and reflect on the food.“ (Judge 6)
Script 3	”Perhaps it is important to say, after mentioning ‘Cosmetic Additives,’ something like: 'that serve to beautify food.' It may give the impression that cosmetics are used (foundation, powder, mascara), since this term is better known for this class of products.“ (Judge 1) ”I think it would be equally important to address the impacts of UPF on the environment and culture.“ (Judge 3) On the characteristics of ultraprocessed foods: ”I thought saying that 'it allows them to be consumed in a practical way at any time of the year' sounded very positive. I think something like: '(...) allows them to be consumed without having to pay much attention, while walking, driving, watching TV, etc.’ Then, in the illustration, instead of showing all the characters eating ultraprocessed foods on the plate, include people eating cereal bars while driving, instant noodles while on the computer, etc.“ (Judge 4) ”Insert something that leads the audience to review food and its accessibility.“ (Judge 6)

Based on the validated scripts for the animated videos, the content approach was divided into the following segments:

1. Introduction to the concept of food processing levels before they are purchased, prepared, and consumed and how these levels influence the quality of the food, whether in a positive way, making it more durable or facilitating preparation, or in a negative way, making the food nutritionally unbalanced and high in calories
2. Deepening the categories and food identification according to the NOVA classification system
3. An approach to UPFs and reports of some of their impacts on health

The videos were structured considering multimedia learning instructional principles. Thus, this production complied with the Coherence Principle, using only pictograms, videos, and narration to fulfill the communication purpose. To meet the Signaling Principle, chart arrows, symbols, and colors were used to highlight elements and topics during the videos. The use of animation and narration only, without adding text or subtitles, respected the Redundancy Principle. The Spatial Contiguity Principle and Temporal Contiguity Principle were respected when relating pictograms and texts in the same scene and when narrating and presenting the pictograms simultaneously, respectively.

The Segmentation Principle and Pre-training Principle were also respected, since the distribution of content was divided into topics and, with each video, the theme was deepened, and terms and concepts were reinforced. The Multimedia Principle was met by opting for the use of words and images. The Modality Principle was not fully met, since, despite the pictograms and videos being associated with the narration, at certain times, the use of technical terms was necessary; therefore, it was decided to add words to the scenes. To meet the Personalization Principle, Voice Principle, and Image Principle, simple and informal language was adopted, narrated by a human voice, without the narrator's exposure. The principles used in each animated video can be accessed in Multimedia Appendix 6.

Once the videos were created, the validation process began. This stage involved 3 specialists in multimedia production, aged between 22 years and 52 years, with an average experience of 6.3 (SD 3.2) years. The results referring to this evaluation are presented in Table 3. The SAM, an instrument used by these specialists, evaluates educational materials and aspects related to its adequacy and effectiveness of communication [23].

**Table 3:** Multimedia production specialists' agreement regarding the adequacy of animated videos (n=3).

Adequacy of material^a^	Animated video 1			Animated video 2			Animated video 3		
	E^b^ (%)	S^c^ (%)	%A^d^	E (%)	S (%)	%A	E (%)	S (%)	%A
Content	100	0	1	100	0	1	91.7	8.3	1
Language	80	20	1	93.3	6.7	1	73.3	26.7	1
Illustrations	100	0	1	100	0	1	100	0	1
Layout and presentation	77.8	22.2	1	77.8	22.8	1	66.7	33.3	1
Stimulation and motivation to learn	88.9	11.1	1	77.8	22.8	1	88.9	11.1	1
Cultural adequacy	100	0	1	83.3	16.7	1	83.3	16.7	1
S-CVI/Ave^e^	—^f^	—	1	—	—	1	—	—	1

^a^Adequacy of the material determined using the average of the items distributed in the 6 domains of the instrument.

^b^E: excellent.

^c^S: suitable.

^d^%A: percentage of agreement.

^e^S-CVI/Ave: scale-level content validity index, averaging method.

^f^Not applicable.

As documented in Table 3, the I-CVI and S-CVI/Ave for the 3 animated videos were equal to 1.00. These results attest to the adequacy and quality of the 3 animated videos in communicating the addressed content.

After being evaluated by specialists in multimedia production, the videos underwent adjustments and finalization and, subsequently, were evaluated by adult representatives of the target audience, proceeding to the last stage of validation.

This stage involved 15 Brazilian adults (6 men and 9 women) with an average age of 37.1 (SD 11.2) years who were residents of 2 states and regions of the country. Regarding race, 1 (7%) participant declared him/herself to be Black, 7 (47%) declared themselves to be Brown, and 7 (47%) were White. With regard to education, 1 (7%) participant declared not having completed basic education, 3 (20%) declared having completed primary education, 6 (40%) had completed secondary education, and 5 (33%) graduated. All of them evaluated the material on a smartphone.

The results of the evaluation of the adequacy of the videos by the target audience are presented in Table 4. The 3 animated videos were considered adequate in terms of content, language, illustrations, layout and presentation, stimulation and motivation to learn, and cultural adequacy. All items had an I-CVI of 1.00, and therefore, the S-CVI/Ave was equal to 1.0. Additional information on the results presented in Tables 1, 3, and 4 has been inserted in Multimedia Appendix 7.

**Table 4:** Assessment of material suitability by representatives of the target audience (n=15).

Adequacy of material^a^	Animated video 1				Animated video 2				Animated video 3		
	E^b^ (%)	S^c^ (%)	%A^d^	E (%)		S (%)	%A	E (%)		S (%)	%A
Content	96.7	3.3	1	98.3		1.7	1	98.3		1.7	1
Language	94.7	5.3	1	96		4	1	94.6		5.4	1
Illustrations	100	0	1	97.8		2.2	1	95.6		4.4	1
Layout and presentation	91.1	8.9	1	97.7		2.3	1	97.8		2.2	1
Stimulation and motivation to learn	97.8	2.2	1	93.3		6.7	1	97.8		2.2	1
Cultural adequacy	100	0	1	100		0	1	100		0	1
S-CVI/Ave^e^	—^f^	—	1	—		—	1	—		—	1

^a^Adequacy of the material as determined using the average of the items distributed in the 6 domains of the instrument.

^b^E: excellent.

^c^S: suitable.

^d^%A: percentage of agreement.

^e^S-CVI/Ave: scale-level content validity index, averaging method.

^f^Not applicable.

This evaluation stage showed that the videos met their purpose of communicating the subject and that they were suitable for what they set out to do, as described in the following participant quotes:

The material was very well prepared.

Participant 14

I thought it was very well explained and done.

Participant 4

(...) I learned a lot, a lot of things I did not imagine (...) I thought the idea was fantastic.

Participant 11

In addition to giving their opinion on the material itself, participants were asked to comment on aspects that could improve the strategy for communicating the content or improve the final quality of the material:

Addressing some diseases triggered by the frequent use of these ultraprocessed foods, but in the form of curiosity.

Participant 5 about animated video 2

Extra material explaining where xanthan gum and other ingredients used to modify flavor, texture, etc. come from.

Participant 9 about animated video 3

Make more videos.

Participant 1 about animated video 3

As far as Participant 5's comment is concerned, this approach is taken in animated video 3, in which the focus is on the characteristics of UPFs and their implications for health. The comments made by Participants 1 and 9, on the other hand, point to a demand that was not covered in the proposal for this work, but which opens up space for the development and dissemination of new educational materials on this subject.

An interesting note in the evaluation of the videos concerns the stimulation or excitement of watching them and reinforces the importance of using this material in contextualized actions.

Excitement/stimulation is also relative. The viewer of the material may not be having a good day or may not be interested in the subject.

Participant 9

Once this last stage of validation was completed, the animated videos were considered finished.

## Discussion

### Principal Findings

Based on intersectoral actions, the Promotion of Adequate and Healthy Eating (abbreviated in Portuguese as PAAS) aims to support individuals and communities to adopt appropriate dietary practices for biological, sociocultural, and environmental aspects [30]. Thus, this study presents the production and validation process of a series of videos that aim to contribute to the expansion of these actions by providing adequate educational resources for FNE actions.

The content validation by the specialists assessed the representativeness of the material in adequately addressing what is proposed, as well as its quality, and increases the likelihood of successful and effective understanding of the content addressed [20]. The evaluations obtained in this stage, even with suggestions for improvement of the message approach, considered the content adequate (according to the CVI), meeting the objective of verifying the cohesion and coherence of the developed scripts.

With regard to the transmission of the educational message, a video has a greater ability to capture the user’s attention, in addition to being more dynamic than folders and booklets, for example [11]. However, to arouse interest and engagement, the educational video needs an effective design to facilitate the communication of its content and promote the audience's understanding. In this sense, during the material creation, special care was taken with the main terms and titles, as well as the exclusion of unnecessary elements, resulting in videos, according to experts, that were didactic and that served the purpose of communicating about the subject.

An educational resource needs to establish interactivity with its audience; therefore, the evaluation by representatives of the target audience was essential to increase the probability of success and effectiveness during educational actions.

It is noteworthy that the use of videos for educational activities has shown promising results in different areas of health [31-33]. According to Adam and collaborators [34], due to growing investment, the production of video content is a means for disseminating educational health content that will probably remain perennial for several years. Besides, this type of resource can influence a new generation of interventions that are more aligned with the needs and contexts of the target audience.

Meppelink and collaborators [11] concluded that animated visual information combined with narration is the best way to communicate complex health messages to people with low health literacy while also being suitable for people with higher levels of health literacy.

The planning stage of this study was important to analyze key aspects of the content approach related to the NOVA classification and DGBP developed so far [1]. In developing the instructional materials, care was taken to use language that communicated the content in a simple and clear manner to make knowledge acquisition effective. Aware that this effectiveness is related to the structure and way of approaching the content, recommendations from the CTML were taken into account in the development of this series of animated videos, applying principles to minimize the cognitive demands on the target audience in processing the topic covered.

Online communication and the use of multimedia educational resources have gained importance in recent years, especially since the COVID-19 pandemic led to new demands in the field of health promotion and surveillance, changing and expanding the strategies of community and individual communication [35]. Given that aspects of this approach to health persist and tend to expand, this type of educational resource is a promising strategy for FNE.

In this context, this study contributes to Promotion of Adequate and Healthy Eating by providing validated animated videos that address the NOVA classification of foods in simplified language while also contributing to the dissemination of the DGBP message by suggesting contextualization of the use of this material that incorporates other recommendations of this guideline [1,36-38].

To date, we have found no publications on studies in which animated educational videos based on NOVA have been developed and validated for use in FNE, so this study appears to be the first with this intention.

A limitation of this study was the lack of a health literacy assessment of the representatives of the target audience to determine the level of understanding of the content. Nevertheless, they were evaluated by 15 individuals with varying levels of education, and all received excellent ratings for language, illustrations, layout, presentation, cultural appropriateness, and motivation to learn. Another limitation concerns the representativeness of the target audience. Although suggestions from the content specialists were accepted to incorporate and expand examples that represented different regions of the country, the animated videos were evaluated by residents of 2 Brazilian regions. Furthermore, adaptations to suit the country's different cultures and eating habits are encouraged.

Finally, the material produced and validated in this study can be used in analytical studies and clinical trials, as well as in actions in primary health care, in person or via mobile health, through platforms or social media. Suggestions for use in workshops and educational actions can be found in a manual developed by the team, available free online [36]. The videos can also be accessed on YouTube by any user of the platform searching for and interested in the topic.

### Conclusion

Animated videos based on the NOVA classification were considered valid in terms of content and suitability for Brazilian adults. It is hoped that they can contribute to the quality of FNE actions, they meet the educational requirements on the subject, and they influence healthy eating practices. We believe that the methodology used in this study to develop these multimedia programs can be explored and used to create new resources related to FNE and in health promotion. It is recommended that additional multimedia resources be developed based on the other chapters and recommendations of the DGBP.

## Appendix

Multimedia Appendix 1 Educational Content Validation Instrument in Health.

Multimedia Appendix 2 Instrument Suitability Assessment of Materials.

Multimedia Appendix 3 Animated video 1.

Multimedia Appendix 4 Animated video 2.

Multimedia Appendix 5 Animated video 3.

Multimedia Appendix 6 Instructional principles used in the animated videos.

Multimedia Appendix 7 Agreement between content and multimedia specialists and representatives of the target audience.

## Abbreviations

CTML: cognitive theory of multimedia learning
CVI: content validity index
DGBP: Dietary Guidelines for the Brazilian Population
ECVIH: Educational Content Validation Instrument in Health
FNE: food and nutrition education
I-CVI: item-level content validity index
LILACS: Literatura Latino-Americana e do Caribe em Ciências da Saúde
NCD: noncommunicable disease
S1: script for animated video 1
S2: script for animated video 2
S3: script for animated video 3
SAM: Suitability Assessment of Materials
S-CVI/Ave: scale-level content validity index, average calculation method
UPF: ultraprocessed foods

## Data Availability

The data sets analyzed during this study are available from the corresponding author on reasonable request.
