# Supplementary material for: Animated Videos Based on Food Processing for Guidance of Brazilian Adults: Validation Study
Source: Interact J Med Res. 2023 Sep 11;12:e49092. doi: 10.2196/49092 (PMC10520766; doi:10.2196/49092)
Supplement: Multimedia Appendix 1 [file ijmr_v12i1e49092_app1.docx]

Educational Content Validation Instrument in Health by Leite et al [20].

| **Objectives:** purposes, goals, or targets |
| --- |
| 1. Contemplates the proposed theme |
| 2. Suits the teaching-learning process |
| 3. Clarifies doubts on the addressed theme |
| 4. Provides reflection on the theme |
| 5. Encourages behavior change |
| **Structure/presentation:** organization, structure, strategy, consistency, and sufficiency |
| 6. Language appropriate to the target audience |
| 7. Language appropriate to the educational material |
| 8. Interactive language, enabling active involvement in the educational process |
| 9. Correct information |
| 10. Objective information |
| 11. Enlightening information |
| 12. Necessary information |
| 13. Logical sequence of ideas |
| 14. Current theme |
| 15. Appropriate text size |
| **Relevance:** significance, impact, motivation, and interest |
| 16. Encourages learning |
| 17. Contributes to knowledge in the area |
| 18. Arouses interest in the theme |
