# Supplementary material for: Animated Videos Based on Food Processing for Guidance of Brazilian Adults: Validation Study
Source: Interact J Med Res. 2023 Sep 11;12:e49092. doi: 10.2196/49092 (PMC10520766; doi:10.2196/49092)
Supplement: Multimedia Appendix 2 [file ijmr_v12i1e49092_app2.docx]

Instrument Suitability Assessment of Materials by Sousa et al [23].

| **Content** |
| --- |
| 1. The purpose is evident |
| 2. Content is about behaviors |
| 3. Content is purpose-focused |
| 4. Content highlights key points |
| **Language** |
| 5. Reading level |
| 6. Uses active voice writing |
| 7. Uses vocabulary with common words in the text |
| 8. Context comes before new information |
| 9. Learning is facilitated by topics |
| **Illustrations** |
| 10. The purpose of the illustration referring to the text is clear |
| 11. Types of illustrations |
| 12. The figures/illustrations are relevant |
| **Layout and presentation** |
| 13. Characteristic of the layout |
| 14. Size and font |
| 15. Subheadings are used |
| **Stimulation/ Motivation of learning** |
| 16. Uses interaction |
| 17. The guidelines are specific and give examples |
| 18. Motivation and self-efficacy |
| **Cultural adequacy** |
| 19. It is similar to your logic, language and experience |
| 20. Cultural figure and examples |
