# Supplementary material for: Animated Videos Based on Food Processing for Guidance of Brazilian Adults: Validation Study
Source: Interact J Med Res. 2023 Sep 11;12:e49092. doi: 10.2196/49092 (PMC10520766; doi:10.2196/49092)
Supplement: Multimedia Appendix 6 [file ijmr_v12i1e49092_app6.docx]

Table S1. Instructional principles and their characteristics

| Acronym | Instructional Principle | What does it characterize? |
| --- | --- | --- |
| P1 | Coherence Principle | Exclusion of unnecessary words, symbols, images, and sounds |
| P2 | Signaling Principle | Highlighting key elements of the content covered |
| P3 | Redundancy Principle | Use of animation and narration instead of animation, narration, and on-screen text |
| P4 | Spatial Contiguity Principle | Use of spatially close pictograms and texts |
| P5 | Temporal Contiguity Principle | Pictograms and narration are presented simultaneously |
| P6 | Segmenting Principle | Content presented in segments |
| P7 | Pre-training Principle | Apresentação geral do conteúdo, antes de detalhá-lo |
| P8 | Modality Principle | Use of pictograms and narration instead of pictograms and text |
| P9 | Multimedia Principle | Use of words and images instead of just words |
| P10 | Personalization Principle | Use of conversational style words instead of formal style |
| P11 | Voice Principle | Use of human voice narration |
| P12 | Image Principle | The image of the narrator is dispensable |

Table S2. Principles adopted in the animated videos

| Principle | Animated Video 1 | Animated Video 2 | Animated Video 3 |
| --- | --- | --- | --- |
| P1 | R | R | R |
| P2 | R | R | R |
| P3 | PR | R | PR |
| P4 | R | R | R |
| P5 | R | R | R |
| P6 | R | R | R |
| P7 | R | PR | R |
| P8 | R | R | R |
| P9 | R | R | PR |
| P10 | R | R | R |
| P11 | R | R | R |
| P12 | R | R | R |

R – Respected: when the principle was completely respected throughout the vídeo

PR – Partially respected: when in some moments of the video it was not possible to respect the principle

NA – Not adopted: when the principle was not adopted in the video
