# Supplementary material for: Animated Videos Based on Food Processing for Guidance of Brazilian Adults: Validation Study
Source: Interact J Med Res. 2023 Sep 11;12:e49092. doi: 10.2196/49092 (PMC10520766; doi:10.2196/49092)
Supplement: Multimedia Appendix 7 [file ijmr_v12i1e49092_app7.docx]

Multimedia Appendix 7

Experts' agreement regarding the content of the scripts

| **Items** | | **Script 1** | | | | **Script 2** | | | | **Script 3** | | | |
| --- | --- | --- | --- | --- | --- | --- | --- | --- | --- | --- | --- | --- | --- |
| 1 | Contemplates the proposed theme | **A** | **D** | **% A** | **I-CVI** | **A** | **D** | **% A** | **I-CVI** | **A** | **D** | **% A** | **I-CVI** |
| 2 | Suits the teaching-learning process | 7 | 0 | 100,0 | 1,00 | 6 | 1 | 85,7 | 0,86 | 7 | 0 | 100,0 | 1,00 |
| 3 | Clarifies doubts on the addressed theme | 7 | 0 | 100,0 | 1,00 | 7 | 0 | 100,0 | 1,00 | 7 | 0 | 100,0 | 1,00 |
| 4 | Provides reflection on the theme | 7 | 0 | 100,0 | 1,00 | 7 | 0 | 100,0 | 1,00 | 7 | 0 | 100,0 | 1,00 |
| 5 | Encourages behavior change | 6 | 1 | 85,7 | 0,86 | 7 | 0 | 100,0 | 1,00 | 6 | 1 | 85,7 | 0,86 |
| 6 | Language appropriate to the target audience | 5 | 2 | 71,4 | 0,71 | 6 | 1 | 85,7 | 0,86 | 6 | 1 | 85,7 | 0,86 |
| 7 | Language appropriate to the educational material | 7 | 0 | 100,0 | 1,00 | 7 | 0 | 100,0 | 1,00 | 7 | 0 | 100,0 | 1,00 |
| 8 | Interactive language, enabling active involvement in the educational process | 7 | 0 | 100,0 | 1,00 | 7 | 0 | 100,0 | 1,00 | 7 | 0 | 100,0 | 1,00 |
| 9 | Correct information | 6 | 1 | 85,7 | 0,86 | 6 | 1 | 85,7 | 0,86 | 7 | 0 | 100,0 | 1,00 |
| 10 | Objective information | 7 | 0 | 100,0 | 1,00 | 7 | 0 | 100,0 | 1,00 | 7 | 0 | 100,0 | 1,00 |
| 11 | Enlightening information | 7 | 0 | 100,0 | 1,00 | 7 | 0 | 100,0 | 1,00 | 6 | 1 | 85,7 | 0,86 |
| 12 | Necessary information | 7 | 0 | 100,0 | 1,00 | 7 | 0 | 100,0 | 1,00 | 7 | 0 | 100,0 | 1,00 |
| 13 | Logical sequence of ideas | 7 | 0 | 100,0 | 1,00 | 7 | 0 | 100,0 | 1,00 | 7 | 0 | 100,0 | 1,00 |
| 14 | Current theme | 7 | 0 | 100,0 | 1,00 | 7 | 0 | 100,0 | 1,00 | 7 | 0 | 100,0 | 1,00 |
| 15 | Appropriate text size | 7 | 0 | 100,0 | 1,00 | 7 | 0 | 100,0 | 1,00 | 7 | 0 | 100,0 | 1,00 |
| 16 | Encourages learning | 6 | 1 | 85,7 | 0,86 | 7 | 0 | 100,0 | 1,00 | 7 | 0 | 100,0 | 1,00 |
| 17 | Contributes to knowledge in the area | 7 | 0 | 100,0 | 1,00 | 7 | 0 | 100,0 | 1,00 | 7 | 0 | 100,0 | 1,00 |
| 18 | Arouses interest in the theme | 7 | 0 | 100,0 | 1,00 | 7 | 0 | 100,0 | 1,00 | 7 | 0 | 100,0 | 1,00 |
| **S-CVI/Ave** |  | - | - | 96,03 | 0,96 | - | - | 97,62 | 0,98 | - | - | 97,62 | 0,98 |

A: agreement

D: disagreement

% A: percentage agreement

I-CVI: item-level content validity index

S-CVI/Ave: scale-level content validity index, averaging method.

Multimedia production specialists' agreement regarding the adequacy of animated vídeos

| **Items** | | **Animated Video 1** | | | | **Animated Video 2** | | | | **Animated Video 3** | | | |
| --- | --- | --- | --- | --- | --- | --- | --- | --- | --- | --- | --- | --- | --- |
| 1 | The purpose is evident | **A** | **D** | **% A** | **I-CVI** | **A** | **D** | **% A** | **I-CVI** | **A** | **D** | **% A** | **I-CVI** |
| 2 | Content is about behaviors | 3 | 0 | 100,0 | 1,00 | 3 | 0 | 100,0 | 1,00 | 3 | 0 | 100,0 | 1,00 |
| 3 | Content is purpose-focused | 3 | 0 | 100,0 | 1,00 | 3 | 0 | 100,0 | 1,00 | 3 | 0 | 100,0 | 1,00 |
| 4 | Content highlights key points | 3 | 0 | 100,0 | 1,00 | 3 | 0 | 100,0 | 1,00 | 3 | 0 | 100,0 | 1,00 |
| 5 | Reading level | 3 | 0 | 100,0 | 1,00 | 3 | 0 | 100,0 | 1,00 | 3 | 0 | 100,0 | 1,00 |
| 6 | Uses active voice writing | 3 | 0 | 100,0 | 1,00 | 3 | 0 | 100,0 | 1,00 | 3 | 0 | 100,0 | 1,00 |
| 7 | Uses vocabulary with common words in the text | 3 | 0 | 100,0 | 1,00 | 3 | 0 | 100,0 | 1,00 | 3 | 0 | 100,0 | 1,00 |
| 8 | Context comes before new information | 3 | 0 | 100,0 | 1,00 | 3 | 0 | 100,0 | 1,00 | 3 | 0 | 100,0 | 1,00 |
| 9 | Learning is facilitated by topics | 3 | 0 | 100,0 | 1,00 | 3 | 0 | 100,0 | 1,00 | 3 | 0 | 100,0 | 1,00 |
| 10 | The purpose of the illustration referring to the text is clear | 3 | 0 | 100,0 | 1,00 | 3 | 0 | 100,0 | 1,00 | 3 | 0 | 100,0 | 1,00 |
| 11 | Types of illustrations | 3 | 0 | 100,0 | 1,00 | 3 | 0 | 100,0 | 1,00 | 3 | 0 | 100,0 | 1,00 |
| 12 | The figures/illustrations are relevant | 3 | 0 | 100,0 | 1,00 | 3 | 0 | 100,0 | 1,00 | 3 | 0 | 100,0 | 1,00 |
| 13 | Characteristic of the layout | 3 | 0 | 100,0 | 1,00 | 3 | 0 | 100,0 | 1,00 | 3 | 0 | 100,0 | 1,00 |
| 14 | Size and font | 3 | 0 | 100,0 | 1,00 | 3 | 0 | 100,0 | 1,00 | 3 | 0 | 100,0 | 1,00 |
| 15 | Subheadings are used | 3 | 0 | 100,0 | 1,00 | 3 | 0 | 100,0 | 1,00 | 3 | 0 | 100,0 | 1,00 |
| 16 | Uses interaction | 3 | 0 | 100,0 | 1,00 | 3 | 0 | 100,0 | 1,00 | 3 | 0 | 100,0 | 1,00 |
| 17 | The guidelines are specific and give examples | 3 | 0 | 100,0 | 1,00 | 3 | 0 | 100,0 | 1,00 | 3 | 0 | 100,0 | 1,00 |
| 18 | Motivation and self-efficacy | 3 | 0 | 100,0 | 1,00 | 3 | 0 | 100,0 | 1,00 | 3 | 0 | 100,0 | 1,00 |
| 19 | It is similar to your logic, language and experience | 3 | 0 | 100,0 | 1,00 | 3 | 0 | 100,0 | 1,00 | 3 | 0 | 100,0 | 1,00 |
| 20 | Cultural figure and examples | 3 | 0 | 100,0 | 1,00 | 3 | 0 | 100,0 | 1,00 | 3 | 0 | 100,0 | 1,00 |
| **S-CVI/Ave** |  | - | - | 100,0 | 1,00 | - | - | 100,0 | 1,00 | - | - | 100,0 | 1,00 |

A: agreement

D: disagreement

% A: percentage agreement

I-CVI: item-level content validity index

S-CVI/Ave: scale-level content validity index, averaging method.

Assessment of material suitability by representatives of the target audience

| **Items** | | **Animated Video 1** | | | | **Animated Video 2** | | | | **Animated Video 3** | | | |
| --- | --- | --- | --- | --- | --- | --- | --- | --- | --- | --- | --- | --- | --- |
| 1 | The purpose is evident | **A** | **D** | **% A** | **I-CVI** | **A** | **D** | **% A** | **I-CVI** | **A** | **D** | **% A** | **I-CVI** |
| 2 | Content is about behaviors | 15 | 0 | 100,0 | 1,00 | 15 | 0 | 100,0 | 1,00 | 15 | 0 | 100,0 | 1,00 |
| 3 | Content is purpose-focused | 15 | 0 | 100,0 | 1,00 | 15 | 0 | 100,0 | 1,00 | 15 | 0 | 100,0 | 1,00 |
| 4 | Content highlights key points | 15 | 0 | 100,0 | 1,00 | 15 | 0 | 100,0 | 1,00 | 15 | 0 | 100,0 | 1,00 |
| 5 | Reading level | 15 | 0 | 100,0 | 1,00 | 15 | 0 | 100,0 | 1,00 | 15 | 0 | 100,0 | 1,00 |
| 6 | Uses active voice writing | 15 | 0 | 100,0 | 1,00 | 15 | 0 | 100,0 | 1,00 | 15 | 0 | 100,0 | 1,00 |
| 7 | Uses vocabulary with common words in the text | 15 | 0 | 100,0 | 1,00 | 15 | 0 | 100,0 | 1,00 | 15 | 0 | 100,0 | 1,00 |
| 8 | Context comes before new information | 15 | 0 | 100,0 | 1,00 | 15 | 0 | 100,0 | 1,00 | 15 | 0 | 100,0 | 1,00 |
| 9 | Learning is facilitated by topics | 15 | 0 | 100,0 | 1,00 | 15 | 0 | 100,0 | 1,00 | 15 | 0 | 100,0 | 1,00 |
| 10 | The purpose of the illustration referring to the text is clear | 15 | 0 | 100,0 | 1,00 | 15 | 0 | 100,0 | 1,00 | 15 | 0 | 100,0 | 1,00 |
| 11 | Types of illustrations | 15 | 0 | 100,0 | 1,00 | 15 | 0 | 100,0 | 1,00 | 15 | 0 | 100,0 | 1,00 |
| 12 | The figures/illustrations are relevant | 15 | 0 | 100,0 | 1,00 | 15 | 0 | 100,0 | 1,00 | 15 | 0 | 100,0 | 1,00 |
| 13 | Characteristic of the layout | 15 | 0 | 100,0 | 1,00 | 15 | 0 | 100,0 | 1,00 | 15 | 0 | 100,0 | 1,00 |
| 14 | Size and font | 15 | 0 | 100,0 | 1,00 | 15 | 0 | 100,0 | 1,00 | 15 | 0 | 100,0 | 1,00 |
| 15 | Subheadings are used | 15 | 0 | 100,0 | 1,00 | 15 | 0 | 100,0 | 1,00 | 15 | 0 | 100,0 | 1,00 |
| 16 | Uses interaction | 15 | 0 | 100,0 | 1,00 | 15 | 0 | 100,0 | 1,00 | 15 | 0 | 100,0 | 1,00 |
| 17 | The guidelines are specific and give examples | 15 | 0 | 100,0 | 1,00 | 15 | 0 | 100,0 | 1,00 | 15 | 0 | 100,0 | 1,00 |
| 18 | Motivation and self-efficacy | 15 | 0 | 100,0 | 1,00 | 15 | 0 | 100,0 | 1,00 | 15 | 0 | 100,0 | 1,00 |
| 19 | It is similar to your logic, language and experience | 15 | 0 | 100,0 | 1,00 | 15 | 0 | 100,0 | 1,00 | 15 | 0 | 100,0 | 1,00 |
| 20 | Cultural figure and examples | 15 | 0 | 100,0 | 1,00 | 15 | 0 | 100,0 | 1,00 | 15 | 0 | 100,0 | 1,00 |
| **S-CVI/Ave** |  | - | - | 100,0 | 1,00 | - | - | 100,0 | 1,00 | - | - | 100,0 | 1,00 |

A: agreement

D: disagreement

% A: percentage agreement

I-CVI: item-level content validity index

S-CVI/Ave: scale-level content validity index, averaging method.
